# Bibliography

**Published:** 1898-11

**Authors:** 


### Bibliography.

Orthodontia, or Malposition of the Human Teeth: Its Prevention and Remedy. By S. H. Guilford, A.M., D.D.S., Ph.D., Professor of Operative and Prosthetic Dentistry, and Dean of the Philadelphia Dental College, etc. Third edition, revised and enlarged. Press of T. C. Davis & Sons, Philadelphia.

That this is the third edition of this valuable text-book on orthodontia in nine years proves, beyond any words, that its value is thoroughly established. This edition has "largely been re-written, its size increased by more than twenty pages, and some fifty new illustrations introduced. . . . Every chapter has been more or less changed to conform to present knowledge, and three new chapters introduced."

The new chapter on "Dynamics of Tooth Movement" will prove a satisfactory addition to students and many practitioners, for the importance of secure anchorage and the proper application of force are the main difficulties to be considered in all cases of regulating.

This work of Dr. Guilford has been reviewed in previous editions, and the opinions heretofore expressed remain in force with the present issue. Its convenient size, systematic treatment of this difficult subject, combined with clearness of description, make it one of the most reliable text-books issued. This was to be expected from the long experience of the author as teacher and practitioner; but, unfortunately, the ability to prepare a good text-book is not always coexistent with the faculty of manipulative skill. The author combines both qualities, and his work, therefore, appeals to all grades of intelligence in dentistry as a work for continued reference and for practical application.

ANATOMY AND HISTOLOGY OF THE MOUTH AND TEETH. By I. N. Broomell, D.D.S. P. Blakiston Son & Co., Philadelphia.

Dr. Broomell's new book will be issued in a few days. The writer has had the privilege of glancing over the advanced sheets, and he is confident he hazards nothing in making the prediction that this will be regarded as the most original book issued in recent years upon the subjects treated. A full review will be given in the December number.

